# Case Report: Immune-Related Toxicity During Adjuvant Treatment With BRAF Plus MEK Inhibitors in a Melanoma Patient

**DOI:** 10.3389/fimmu.2020.579523

**Published:** 2020-11-17

**Authors:** Andrea Boutros, Chiara Schiavi, Federica Cecchi, Francesco Spagnolo, Antonio Guadagno, Enrica Teresa Tanda, Francesca Giusti, Giuseppe Murdaca, Paola Queirolo

**Affiliations:** ^1^Department of Medical Oncology, U.O. Oncologia Medica 2, IRCCS Ospedale Policlinico San Martino, Genoa, Italy; ^2^Department of Internal Medicine and Medical Specialties (DiMI), School of Medicine, University of Genoa, Genoa, Italy; ^3^Clinical Immunology Unit, Department of Internal Medicine, University of Genoa, Ospedale Policlinico San Martino, Genoa, Italy; ^4^Anatomic Pathology Unit, IRCCS Ospedale Policlinico San Martino, Genoa, Italy; ^5^Department of Internal Medicine, University of Genoa, Policlinic San Martino Hospital, Genoa, Italy; ^6^Division of Medical Oncology for Melanoma, Sarcoma, and Rare Tumors, IEO, European Institute of Oncology IRCCS, Milan, Italy

**Keywords:** melanoma, dabrafenib and trametinib, BRAF and MEK targeted therapy, immune-related adverse event irAE, immunotherapy, sarcoidosis, tattoo, uveitis

## Abstract

Adjuvant treatment of operated melanoma has deeply changed in the last few years with the introduction of immune-checkpoint inhibitors and BRAF/MEK inhibitors. Sarcoidosis is a systemic inflammatory disease causing non-caseous granulomatous reactions. Sarcoid-like granulomatous reactions have been reported in patients with advanced melanoma, mostly related to immunotherapy with immune-checkpoint inhibitors. We report a case of a 38-year-old woman with stage III operated melanoma treated with adjuvant BRAF plus MEK inhibitors, who developed sarcoidosis-like syndrome with systemic involvement, resolved after discontinuation of treatment. The occurrence of immune-related toxicity with the use of MAPK inhibitors supports the hypothesis that this class of drugs may also have an immunological effect, and that the long-term efficacy of adjuvant MAPK inhibitors may be due to their immunological function.

## Introduction

The introduction of adjuvant BRAF and MEK inhibitors for patients with resected stage III *BRAF* V600-mutant melanoma has led to a 49% reduction of the risk of recurrence compared with placebo, with a 5-year relapse-free survival of 52% (1, 2). Thus, these results suggest that not only immunotherapy but also targeted therapy can achieve a long-term benefit, even after the end of the treatment. In fact, BRAF plus MEK inhibitors may also have an immunological function (3). These immune effects can play a role in terms of both efficacy and toxicity. Here we report a case of a patient with resected melanoma who developed systemic sarcoidosis-like syndrome following adjuvant therapy with BRAF plus MEK inhibitors.

## Case Report

A 38-year old female patient was diagnosed with cutaneous melanoma of the back in 2019 (pT2a pN1a M0 according to AJCC 8^th^ edition staging system). Her past medical history was unremarkable, except for De Quervain’s subacute thyroiditis about 10 years earlier. Her family history was relevant solely to systemic lupus erythematosus in her maternal grandmother.

**Figure 1:**
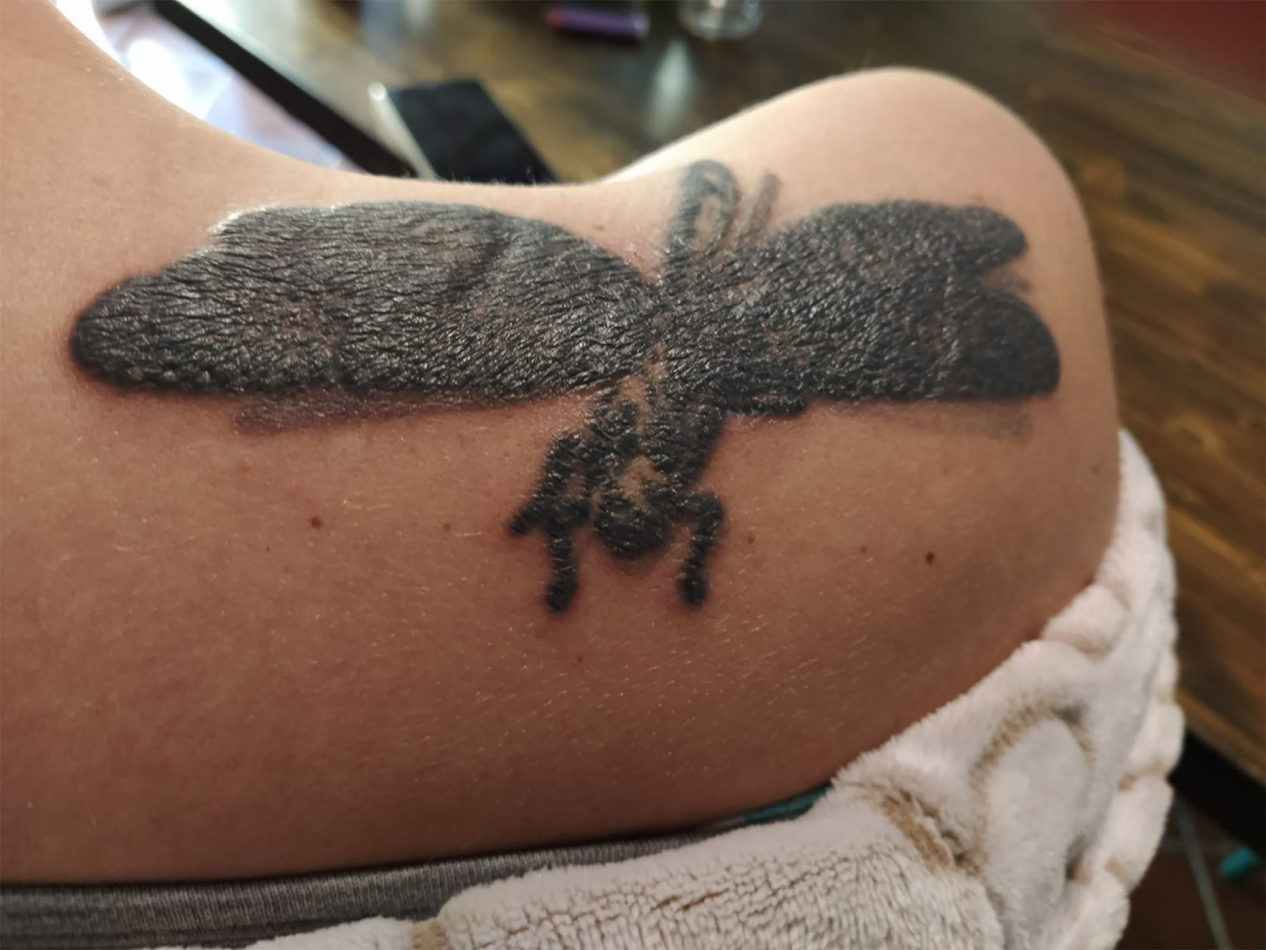
Fifteen year-old tattoo made of black pigment with erythematous and painful infiltrative reaction with papules and plaques.

The molecular analyses revealed the *BRAF* V600E mutation, and adjuvant therapy with dabrafenib plus trametinib was started in July 2019. Treatment was poorly tolerated from the very beginning due to incoercible fever. The fever was poorly responsive both to treatment withdrawal and to paracetamol (unlike the normal course of this type of adverse event). After 3 months from the start of dabrafenib plus trametinib, the patient complained an inflammatory reaction of her tattoos (**Figure 1**), which was clinically swollen, erythematous and painful, in a *scar sarcoidosis*-like reaction. This tattoo reaction improved on topical corticosteroids. On laboratory tests, there was an increase in transaminase, alkaline phosphatase (ALP) and gamma glutamyl transferase (γGT) levels. Therefore, serological tests for HAV, HBV, HCV, EBV, CMV hepatotropic viruses infections were performed with negative results; autoimmunity tests revealed elevated angiotensin-converting enzyme (ACE) and a 1:160 title of the antinuclear antibody (ANA) with nucleolar pattern. The C-reactive protein (CRP) value was 16.4 mg/L [normal range 0.0–5.0 mg/dl].

A few days later, the patient was admitted to the ophthalmologic emergency department with blurred vision, eye pain and eye redness, and was diagnosed with anterior granulomatous synechizing uveitis, suggesting a drug-related uveitis. Treatment with dabrafenib plus trametinib was temporarily interrupted, and the uveitis was treated with eye drops corticosteroids.

**Figure 2:**
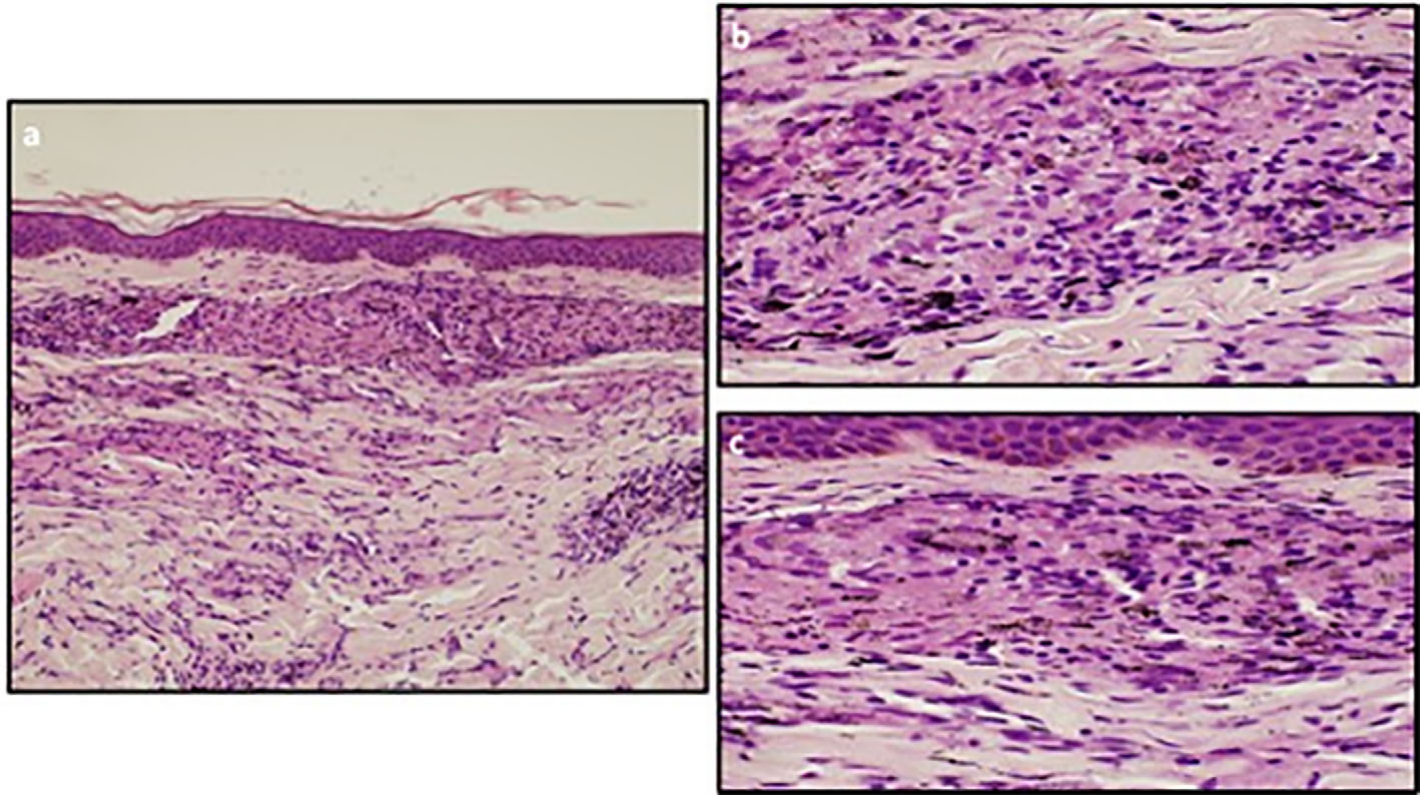
**(A)** The skin biopsy shows a sarcoidal granulomatous dermatitis within the dermis. The epidermis is normal. (Ematoxylin and Eosin staining; 10x). **(B, C)** The sarcoidal granulomas are discrete nodular aggregates of epithelioid histiocytes with very few lymphoid cells at the periphery (“naked granulomas”). Tattoo pigment is seen within the cytoplasm of histiocytes. Necrosis is not present. (Ematoxylin and Eosin staining; 40x).

**Figure 3:**
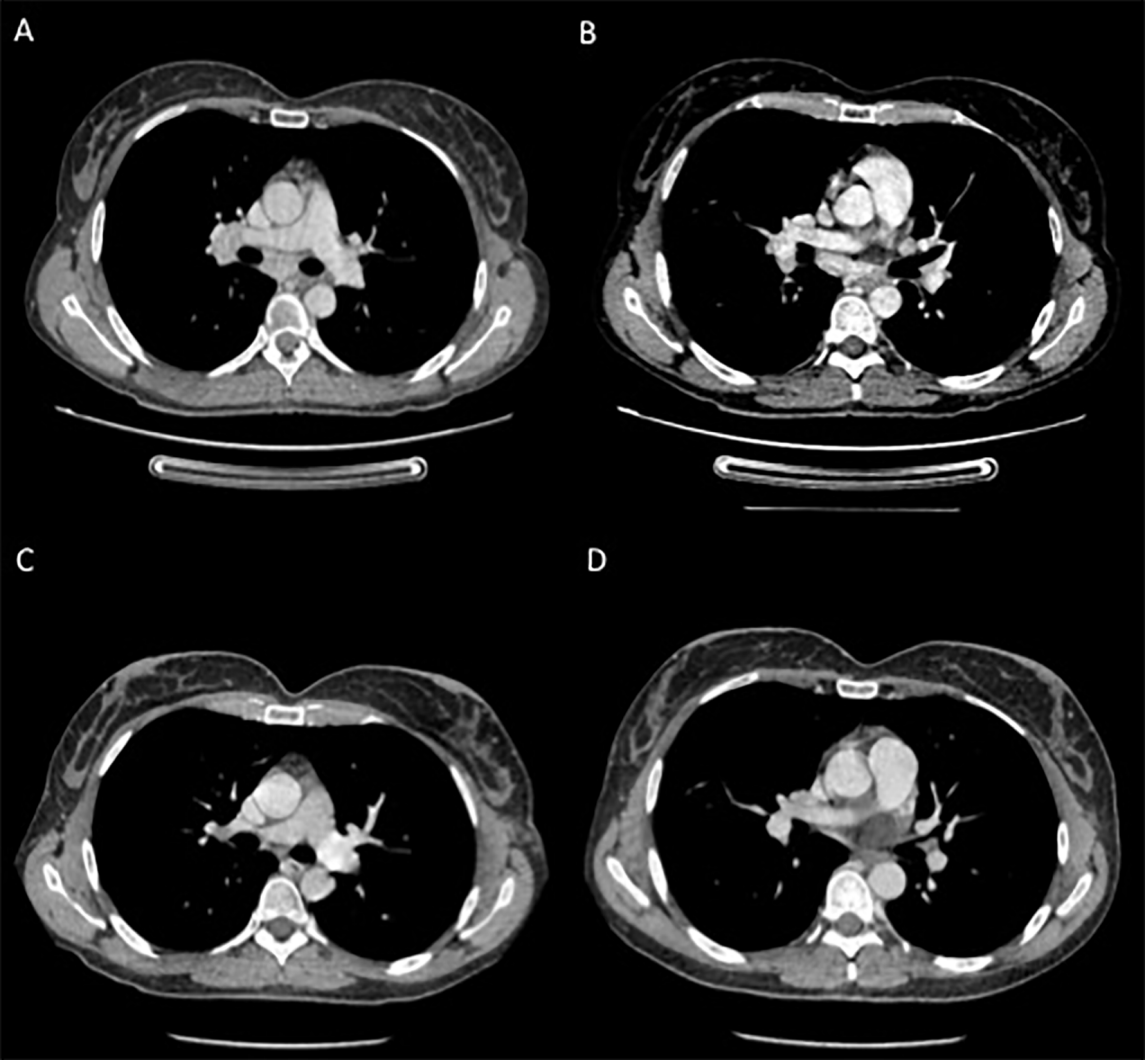
Contrast CT-scans performed during targeted therapy **(A, B)** showing enlarged mediastinal lymph nodes; and **(C, D)** after the definitive suspension of treatment showing reduction in the diameter of the previously reported lymph nodes.

On withdrawal of both dabrafenib and trametinib for a longer period of time, the signs and symptoms (fever, tattoo inflammation, elevated transaminases and uveitis) gradually improved. The CT-scan assessment showed enlarged mediastinal lymph nodes with inflammatory characteristics with no signs of disease relapse (**Figure 3**). One month after treatment discontinuation, dabrafenib plus trametinib was resumed with a dose reduction. However, treatment restart resulted in the relapse of all symptoms, first and foremost the sarcoid-like reaction of the tattoos and the persistent fever. Therefore, a skin biopsy was taken with the pathology report showing non necrotizing granulomatous lesions in the dermal layer, consisting of epithelioid histiocytes incorporating blackish pigment (attributable to tattoo) and giant pluri-nucleated cells, defined as “Langhans cells” (**Figure 2**). The histochemical research of hyphae, fungal spores and acid-alcohol resistant bacilli was negative. Therefore, the final diagnosis of non-caseous granuloma, coherent with sarcoidosis, was made. After this finding, the adjuvant treatment was permanently discontinued after a total of 6 months of treatment.

At the last CT scan reassessment, the patient remained disease-free, and the mediastinal lymph nodes were no longer enlarged (**Figure 3**). The patient is currently asymptomatic, with no signs of melanoma recurrence or inflammatory relapse as of the time of writing this letter.

## Discussion

Although cases of BRAF inhibitor-related sarcoidosis-like syndromes (especially cutaneous) have already been reported (4), to our knowledge, this is the first report of a case of BRAF plus MEK inhibitor adjuvant treatment-related systemic sarcoidosis-like syndrome. Sarcoidosis is a multisystemic disorder that can potentially affect any organ. In our case, the diagnosis of systemic sarcoidosis was based on the Valeyre criteria (5): (1) compatible clinical presentation; (2) evidence of non-caseating granuloma; and (3) no alternative diagnosis. In addition, in this case, the course of the clinical manifestations was consensual with dechallenge and rechallenge of treatment with dabrafenib and trametinib, as the complete resolution of the manifestations (such as mediastinal lymph node involvement and tattoo swelling) followed the definitive discontinuation of therapy.

What we observed, as described in other cases, suggests that treatment with dabrafenib and trametinib breaks the immune tolerance to, for example, tattoo ink, unmasking a hypersensitivity reaction (4).

Similar reactions have also been described in the context of immune reconstitution syndrome in patients treated with antiretroviral therapy, where the hypersensitivity reaction was masked by T CD4 lymphopenia (6).

It has also been reported that BRAF inhibitory agents, such dabrafenib and vemurafenib, may induce a paradoxical overactivation of the MAPK pathway, favoring the activation of T lymphocytes (7).

An analysis of clinical and preclinical trials (8) has shown that the combination of BRAF and MEK inhibitors not only has a direct effect on melanoma cells, but also an indirect function through anti-tumor immunity, promoting the formation of an immunostimulating microenvironment, enhancing tumor-infiltrating lymphocytes, improving antigen recognition and T effector cells activity (8).

However, differently from the mentioned cases, in this report we discussed a case of a patient with non-evident disease receiving dabrafenib and trametinib in the adjuvant setting.

In this perspective, it would be interesting to identify biomarkers of immune response activation in patients receiving targeted therapy in this setting. In fact, a recently published biomarker analysis from the COMBI-AD trial showed that the expression of markers of an adaptive immune response, such as tumor mutational burden (TMB) and an IFNγ gene expression signature, may play a role in identifying patients with stage III melanoma who could derive clinical benefit from targeted therapy. In particular, when IFNγ signature is present, it is more likely that an adaptive antitumor immunity has been mounted (9). Adaptive immunity plays a key role particularly in case of high TMB, which could potentially lead to an increased number of molecular escape mechanisms (9).

Nevertheless, further preclinical and clinical investigations are needed to better understand the immunological role of targeted therapy with BRAF and MEK inhibitors as well as to identify the characteristics of patients who may benefit most in this setting.

## Conclusion

In summary, we reported a case of BRAF plus MEK inhibitor adjuvant treatment-related systemic sarcoidosis. The impact of this drug-related reaction on the effectiveness of the treatment is still unclear and needs to be further investigated.

As shown by preclinical findings and by the reported clinical manifestations, BRAF and MEK inhibitors have an immunological activity, reinforcing the hypothesis that the long-term efficacy of adjuvant MAPK inhibitors may be due to their immunological effects.

## Data Availability Statement

The original contributions presented in the study are included in the article/supplementary material. Further inquiries can be directed to the corresponding author.

## Ethics Statement

Written informed consent was obtained from the individual(s) for the publication of any potentially identifiable images or data included in this article.

## Author Contributions

PQ and GM contributed equally to this work as supervisors. AB assisted in patient management, article writing, and review of literature. CS assisted in patient management and review of literature. FC, ET, and FG assisted in patient management and diagnostic workup. AG provided the histopathological figures and captions. FS assisted in article writing and review of literature. All authors contributed to the article and approved the submitted version.

## Conflict of Interest

The authors declare that the research was conducted in the absence of any commercial or financial relationships that could be construed as a potential conflict of interest.
